# The Dihydrouridine landscape from tRNA to mRNA: a perspective on synthesis, structural impact and function

**DOI:** 10.1080/15476286.2022.2078094

**Published:** 2022-05-30

**Authors:** Olivier Finet, Carlo Yague-Sanz, Florian Marchand, Damien Hermand

**Affiliations:** URPHYM-GEMO, The University of Namur, Namur, Belgium

**Keywords:** RNA modification, Dihydrouridine, Dus, epitranscriptome

## Abstract

The universal dihydrouridine (D) epitranscriptomic mark results from a reduction of uridine by the Dus family of NADPH-dependent reductases and is typically found within the eponym D-loop of tRNAs. Despite its apparent simplicity, D is structurally unique, with the potential to deeply affect the RNA backbone and many, if not all, RNA-connected processes. The first landscape of its occupancy within the tRNAome was reported 20 years ago. Its potential biological significance was highlighted by observations ranging from a strong bias in its ecological distribution to the predictive nature of Dus enzymes overexpression for worse cancer patient outcomes. The exquisite specificity of the Dus enzymes revealed by a structure-function analyses and accumulating clues that the D distribution may expand beyond tRNAs recently led to the development of new high-resolution mapping methods, including Rho-seq that established the presence of D within mRNAs and led to the demonstration of its critical physiological relevance.

The RNA backbone is a succession of covalently bound ribonucleotides whose nucleobase, ribose or 5’-extremity can be modified. To date, there are more than 150 known RNA chemical modifications, spanning the three domains of life and viruses. RNA modifying enzymes can work as (cofactor-dependent) stand-alone proteins, be part of a protein complex that is required for modification or are guided by small nucleolar RNAs (snoRNAs). tRNAs are the most heavily modified RNA species with up to 25% (in eukaryotes) and 15% (in prokaryotes) of their ribonucleotides being modified [1]. It is estimated that one-fifth of all known tRNA modifications is spread across all domains of life [2]. Ribosomal RNAs are also widely modified yet to a lesser extent with up to 2% of modified positions [3]. The abundance of rRNAs and tRNAs facilitated their study and the modification status of their building blocks. However, other RNA species have been known to carry post-transcriptional modifications (PTMs) for decades. Besides the well-characterized eukaryotic mRNA 5’-cap, internal modifications are found in coding RNAs, such as the highly abundant m^6^A, which was detected in mRNAs more than forty years ago [4]. In Eukarya, snoRNAs and snRNAs carry 5’-end and internal modifications but have a narrower range of PTMs [5]. Over the past decade, a still-increasing set of modifications including m^6^A, m^6^Am, m^5^C, hm^5^C, ψ, m^1^A or 2’-O-Me was mapped at transcriptome-scale, which has been largely discussed elsewhere, for example [6,7].

## 1. Dihydrouridine is structurally unique

**Figure 1.**
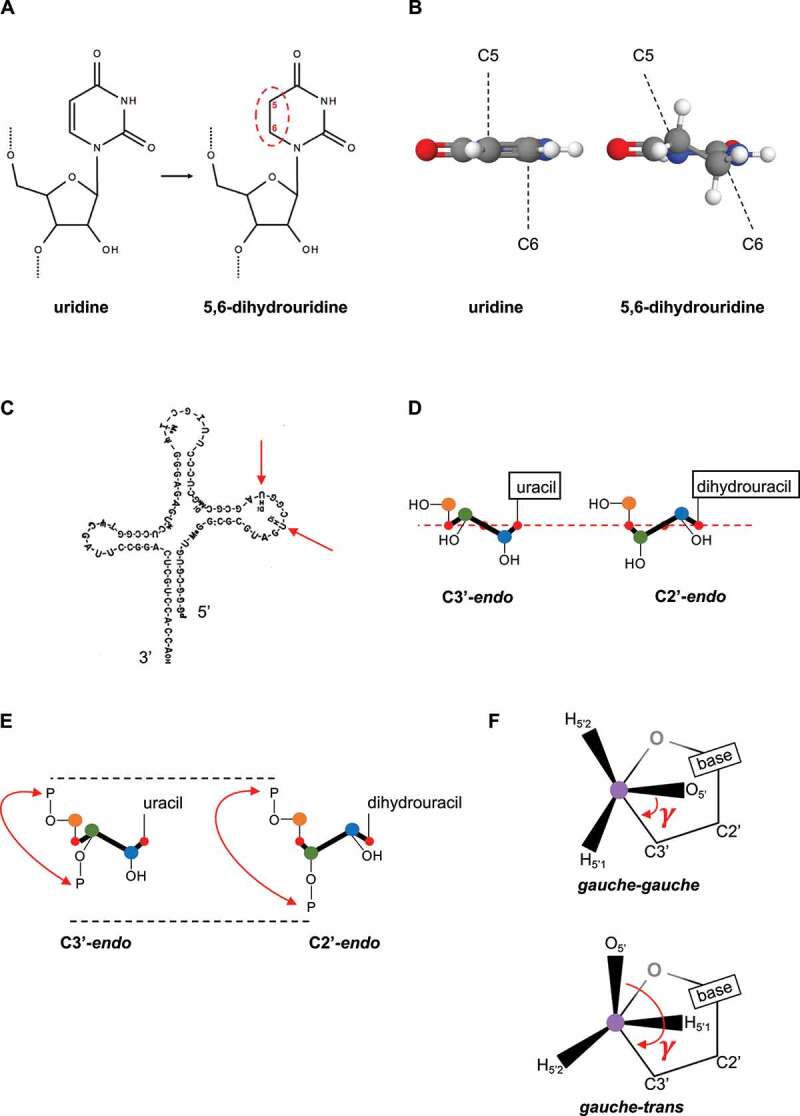
Biochemistry of dihydrouridine.

The dihydrouridine (D, sometimes DHU) RNA modification is a modified pyrimidine nucleoside whose corresponding nucleobase is 5,6-dihydrouracil. D is synthesized from uridine (U) by hydrogenation [8] (Fig. 1A). Reduction of the uridine C5-C6 bond generates a saturated nonplanar and nonaromatic nucleobase that is a landmark of dihydrouridine (Fig. 1B). Although the chemical synthesis of dihydrouracil was already reported in 1896, its first detection in a biological sample dates back from 1952 when it was isolated from the beef spleen [9]. Using *in vitro* approaches, dihydrouridine monophosphate (dihydro-UMP) was shown to be efficiently introduced into RNA molecules, but whether D was a genuine component of cellular RNA was still to be determined [10,11]. D was then reported as a naturally occurring component of yeast tRNA^Ala^ and included in the first published structure of a ribonucleic acid [12,13] (Fig. 1C). Simultaneously to the publication of the tRNA sequence, Visser and colleagues were already discussing the possibility that ‘non-random distribution of hydrogenated pyrimidine [dihydrouridine] may be explained more readily by a process of enzymatic hydrogenation at the polynucleotide level’ ([11], p. 297).

**A** Reduction of uridine into dihydrouridine. **B** Dihydrouracil is a nonplanar nucleobase (carbon in gray, nitrogen in blue, oxygen in red and hydrogen in white). **C** First published structure of a ribonucleic acid (yeast tRNA^Ala^) where D (red arrows) are shown in a loop at the 5’-end [12]. **D** Schematic representation of ribose pucker. C5’ (orange dot) is considered as being *above* the C4’-O’-C1’ plane (red dashed line and red dots). **Left panel**: C3*’-endo* has the C3’ (green dot) above the plane. **Right panel**: C2*’-endo* has the C2’ (blue dot) above the plane. **E** C2’-*endo* pucker produces a longer 5’-phosphate/3’-phosphate distance and therefore spans the polynucleotide [147]. **F** Schematic representation of ribose *gauche-gauche* and *gauche-trans* conformations. C5’ (purple dot) adopts different torsion angles (γ) that modulate the positioning of C5’-bound atoms (H_5ʹ1_,H_5ʹ2_ and O_5’_).

In the next decades, optical studies along with X-ray and NMR crystallographic analyses elucidated the biochemical properties of dihydrouridine. The structures of the nucleobase, the nucleotide, of D-containing tRNAs and D-containing oligoribonucleotides led to the conclusions that; (I) the carbon 6 of the nucleobase (C6) is out of the plane after the C5-C6 double bond reduction, (II) the deviation from the planar nature of the pyrimidine results in the loss of the stacking ability with neighboring nucleobases, (III) the C2’-*endo* conformation is adopted by the ribose moiety whereas canonical ribonucleotides prefers the C3’-*endo* conformation (Fig. 1D) and (IV) the C2’-*endo* pucker is propagated to the 5’-nucleotide (references and comments in Table 1). Therefore, the complete destacking of the bases and the unusual adoption of C2’-*endo* ribose pucker make dihydrouridine a unique modification [14]. The structural properties of dihydrouridine include the potential destabilization of the RNA structure (by promoting C2’-*endo* conformation) and molecular flexibility (by spanning the sugar-phosphate backbone, Fig. 1E). In parallel, the D crystal structure revealed the adoption of *gauche-trans* or *trans-gauche* conformations around the C4’-C5’ ribose bond, rather than the common *gauche-gauche* rotamer (Fig. 1F, references in Table 1). In addition, the dihydrouracil nucleobase was shown to be in *anti* orientation in respect to the ribose moiety (i.e. with C2 = O pointing away from the sugar) (**Movie 1**, references in Table 1). More recently, the contribution of D in folding of the D loop was investigated by NMR, which revealed that the absence of D results in the stable stem-loop hairpin to adopt several undefined interconverting conformations in solution [15].

**Table 1.** Seminal studies on optical and structural properties of dihydro-uracil/-uridine

Structural and optical studies	Comments	References
dihydrouracil	C5-C6 and N1 out of the base plane expectation for Watson-Crick interaction impairment	[140]
dihydrouridine	deviation from planarity *anti* orientation C2’-*endo* conformation	[143]
	*anti* orientation favoured *gauche-trans* and *trans-gauche* conformations	[149]
	deviation from planarity *anti* orientation preference for C3’-*exo* and C2’-*endo* conformations preference for *gauche-gauche* conformation	[85]
	deviation from planarity *anti* orientation C2’-*endo* conformation favoured *gauche-trans* and *trans-gauche* conformations	[142]
	C6 out of the base plane no base stacking and *anti* orientation C2’-*endo* conformation favoured *gauche-trans* and *trans-gauche* conformations	[141]
dinucleoside phosphate: DpA, ApD, GpD	DpA stacking ApD and GpD low stacking	[137]
yeast or *E. coli* tRNAs	formation of a D-loop with increased hydrophobicity D provides extra flexibility interaction of D-loop and TψC-loop	[135,138,139,144–146]
trinucleoside phosphate: Dp(acp^3^U)pA	C2’-*endo* conformation and upstream propagation	150
trinucleoside phosphate: ApDpA	C6 out of the base plane no base stacking enhanced C2’-*endo* conformation at low temperature (5°C) and downstream propagation	[134,, 136]

The table is divided into three columns; (I) studied chemical entities, (II) main conclusions relative to the nucleobase (green writing) or to the ribose (blue writing) and (III) references. C (carbon), N (nitrogen), A (adenosine), D (dihydrouridine), G (guanosine), p (5’-3’ phosphodiester bond), acp^3^U (3-(3-amino-3-carboxypropyl) uridine).

To sum up, D is the sole known non-aromatic modified nucleotide, a feature that promotes noncanonical ribose conformation and hinders proper base stacking. These features have potential deep consequences on many if not all aspects of processes implicating RNA. Indeed, the RNA ‘structurome’ affects splicing, translation or stability [16,17]. In the context of the epitranscriptomic landscape and its readers, it is worth mentioning that the C2′-*endo* conformation promoted by D is bound by RNA recognition motifs (RRM) and that D may therefore enforce the binding of the large repertoire of RNA binding proteins [18].

## 2. Detection of dihydrouridine

Since the discovery of D, several methods to detect this modified nucleotide were developed based on its physicochemical properties. Their recent adaptations to high-throughput sequencing are discussed in a separate section.

### 2.1 Sodium borohydride and alkaline treatments

**Figure 2.**
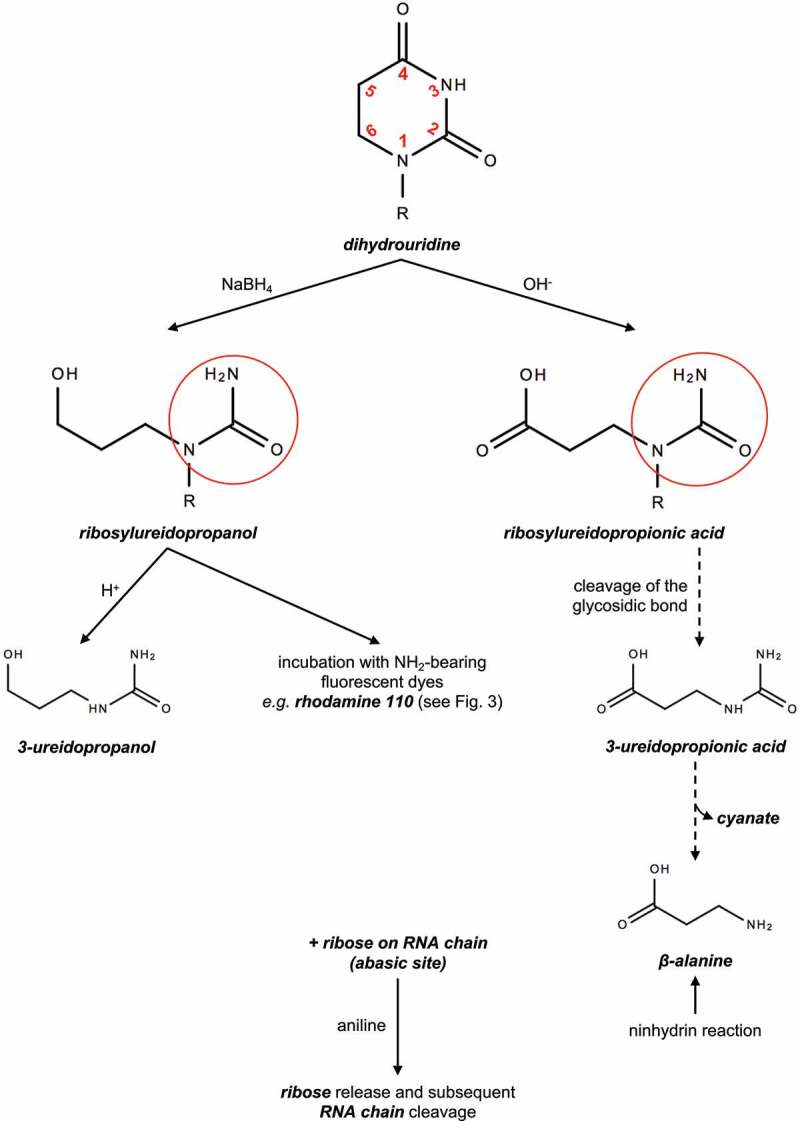
Molecular fate of D upon sodium borohydride (NaBH_4_) or alkaline (OH^−^) treatments.

Distinguishing the RNA modification from its canonical nucleotide is challenging. Chemical treatments affecting D are summarized in Table 2. The dihydrouridine undergoes ring opening upon sodium borohydride (NaBH_4_) or alkaline (OH^−^) treatments, resulting in the formation of an ureido-group (NH_2_CONH) linked to an alcohol or a carboxylic acid, respectively (Fig. 2) [19–21]. The accumulation of ureido-groups can be quantified by a colorimetric assay [22]. The ribosylureidopropanol (D + NaBH_4_) can be used for labelling of RNA with a fluorescent dye or for the cleavage of ureidopropanol upon acid conditions (H^+^) [23–30]. The ribosylureidopropionic acid (D + OH^−^) is used as a semi-quantitative tool following the breakdown between ribose and ureidopropionic acid, this latter being decomposed to β-alanine that serves as a substrate for a colorimetric assay with ninhydrin [31,32]. The D-ring disruption upon OH^−^ condition was also shown to generate an RT (reverse transcription) termination assessed by primer extension [33]. Both treatments, NaBH_4_ or OH^−^ result in an abasic site (nucleobase-free ribose) that leads to the cleavage of the RNA chain with aniline treatment [34,35]. These chemical reactivities of D have been recently exploited to develop transcriptome-wide mapping of D occupancy (see below).

R stands for ribose attached to the RNA chain. Red circles highlight ureido-groups.H^+^ stands for acid conditions. Details in the text.

**Table 2.** Chemical reactions and techniques specifically applicable to D

Principles	tRNA	Comments	References
alkaline hydrolysis	/	sodium hydroxide → cleavage at the N3-C4 linkage of dihydrouracil	[19]
β-alanine detection	Y	sodium hydroxide → D-ring opening → partial β-alanine formation → ninhydrin colorimectric assay	[32]
sodium borohydride reduction	Y	sodium borohydride → cleavage at the N3-C4 linkage of dihydrouridine	[20]
hydrochloric acid hydrolysis	B, Y, M	sodium borohydride → D-ring opening → hydrochloric acid → cleavage of the glycosodic bond	[24,and b; 26]
loss of absorbance	Y	loss of absorbance at 265 nm upon mild sodium hydroxide treatment	[132]
cleavage at D position	Y	sodium borohydride → D-ring opening → aniline treatment → RNA chain cleavage	[34]
ureido-group detection	B, Y, M	sodium hydroxide → D-ring opening → solution neutralization → colorimetric assay after iron chloride addition (550 nm)	[22]
replacement of D by proflavine or EtBr	Y	sodium borohydride → D-ring opening → incubation with the dye	[29]
microarray	Y	differential fluorescent labeling of tRNA from WT and *dus* strains → annealing on a microchip → quantification of A-U *vs* A-D interaction	151
primer extension	B, Y	sodium hydroxide → D-ring opening → assessment of RT termination by primer extension	[23,33]
fluorescent labeling with rhodamine 110	B	*in vitro* tRNA → *in vitro* dihydrouridylation → sodium borohydride → incubation with the dye	[23]
replacement of D by Cy3 or 5	B, Y	sodium borohydride → D-ring opening → incubation with the dye	[27]
benzoyhydrazide addition	B	sodium borohydride → THU formation → incubation with benzohydrazide	[42]
predictive modelling	B, Y	jackknife-based test to predict modified sites	[113,115]
nanopore sequencing	B	RNA through a nanopore scale → specific ionic signature	[116]

The table is divided into four columns; (I) list of principles, (II) phylogenetic origin of the studied tRNA; B (bacterial), Y (yeast), M (mammalian), (III) general comments on the principle. EtBr (ethidium bromide), N (nitrogen), C (carbon), RT (reverse transcription), WT (wild type), *dus* (dihydrouridine synthase gene), A-U (adenine-uridine Watson-Crick interaction), A-D (adenine-dihydrouridine Watson-Crick interaction), Cy3/5 (cyanines 3 or 5), THU (tetrahydrouridine) and (IV) references. Only the seminal works are cited although most of the techniques were implemented in other studies. Chromatographic applications are not listed here.

Thin-layer chromatography was first used to spot ^3^H-labelled D on a cellulose membrane after oxidation with an inorganic salt and reduction with borohydride [36]. In reverse-phase high performance liquid chromatography (HPLC), D is the earliest eluting nucleoside, sometimes barely distinguished from contaminants with short retention times. However, its detection is possible at 254 nm [37] and detectability is improved by lowering the wavelength at 210 or 230 nm [38]. Accurate determination of D is readily obtained by liquid chromatography coupled to mass spectrometry (LC-MS) [37].

### 2.2 Effects on double strand formation

The comparison of binding properties of polyU- *vs* polyD-nucleotides with polyA revealed a reduced ability of polyD to interact with polyA although the reduction of uridine occurs at Hoogsteen edge (C5 = C6) of the nucleobase whereas the hydrogen bonds of the Watson-Crick interactions are formed from positions 3 and 4 (N3-C4 = O) [39]. The decreased binding capacity is nevertheless too weak to induce a clear RT termination in a primer extension context [40]. Nevertheless, Phizicky and colleagues took advantage of the decreased D-A binding to implement a microarray-based technology to monitor the presence of D at specific tRNA positions [33,41].

### 2.3 Labeling with a fluorescent dye

**Figure 3.**
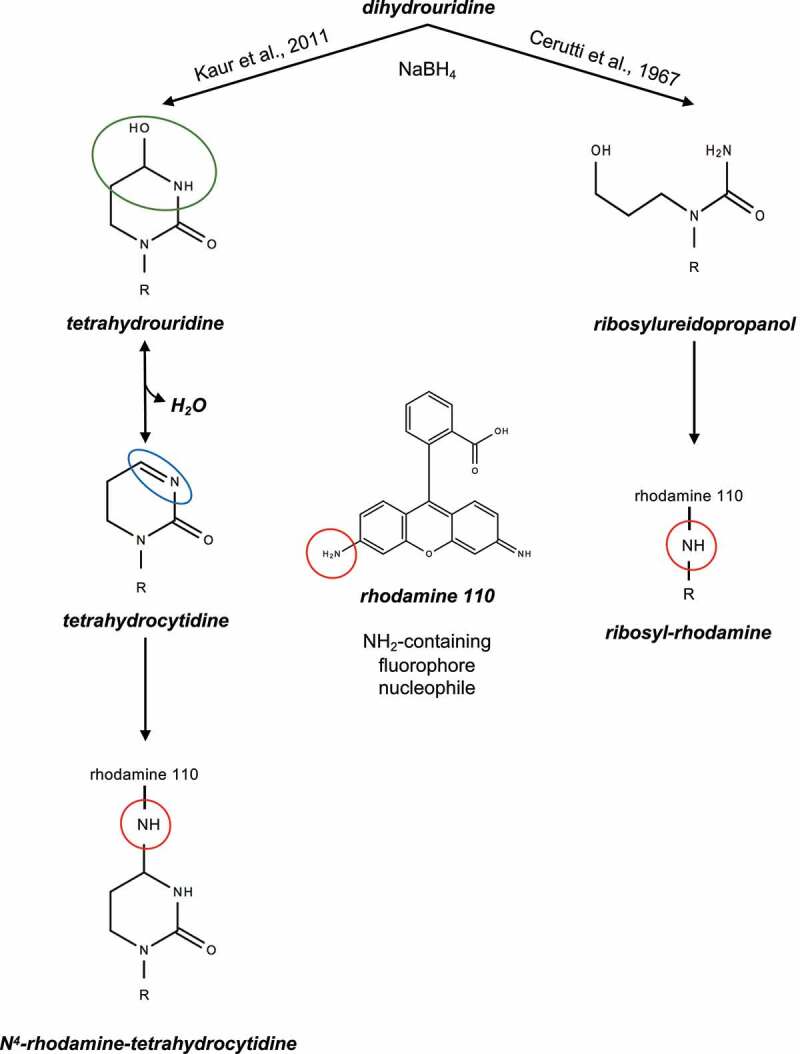
Proposed mechanism of D rhodamine labeling following sodium borohydride (NaBH_4_) reduction.

As stated above, the treatment of D by sodium borohydride is a prerequisite for the subsequent incorporation of fluorescent molecules, and more specifically of NH_2_-dyes (*e.g*. proflavine, rhodamine, cyanine hydrazide). Fig. 2 shows that the D-ring opening forms ribosylureidopropanol in a NaBH_4_-dependent manner. However, this commonly accepted N3-C4 cleavage has been recently challenged [42]. Cooperman and colleagues induced tRNA reduction with sodium borohydride and performed labelling with NH_2_-containing fluorophores nucleophiles. By combining TLC (thin layer chromatography) and mass spectrometry, they detected tetrahydrouridine (THU) instead of the expected ribosylureidopropanol. Based on their results, they proposed that the dihydrouridine C4 carbonyl group is reduced by the H^−^ donor NaBH_4_ to THU. Upon the addition of an NH_2_-dye in acid conditions, a nucleophilic substitution occurs on the C4 hydroxyl group by formation of a Schiff base-bearing intermediate called tetrahydrocytidine (THC) and consecutive fluorophore binding. Fig. 3 summarizes both mechanisms in the context of the addition of the rhodamine 110 fluorophore.

**Left panel**, dihydrouridine is reduced to tetrahydrouridine that is characterized by an electrophilic carbonilamine (green circle), including the C4 hydroxyl group (C-OH). Upon addition of the nucleophilic rhodamine 110 in acid conditions, the tetrahydrocytidine intermediate is formed with its reactive Schiff base (blue circle). Covalent binding of rhodamine 110 occurs by substitution of the C4 hydroxyl group. **Right panel**, ureidopropanol generated by D-ring opening is replaced by rhodamine 110. R stands for ribose attached to the RNA chain.

## 3. Seminal landscape of the distribution of Dihydrouridine

**Figure 4.**
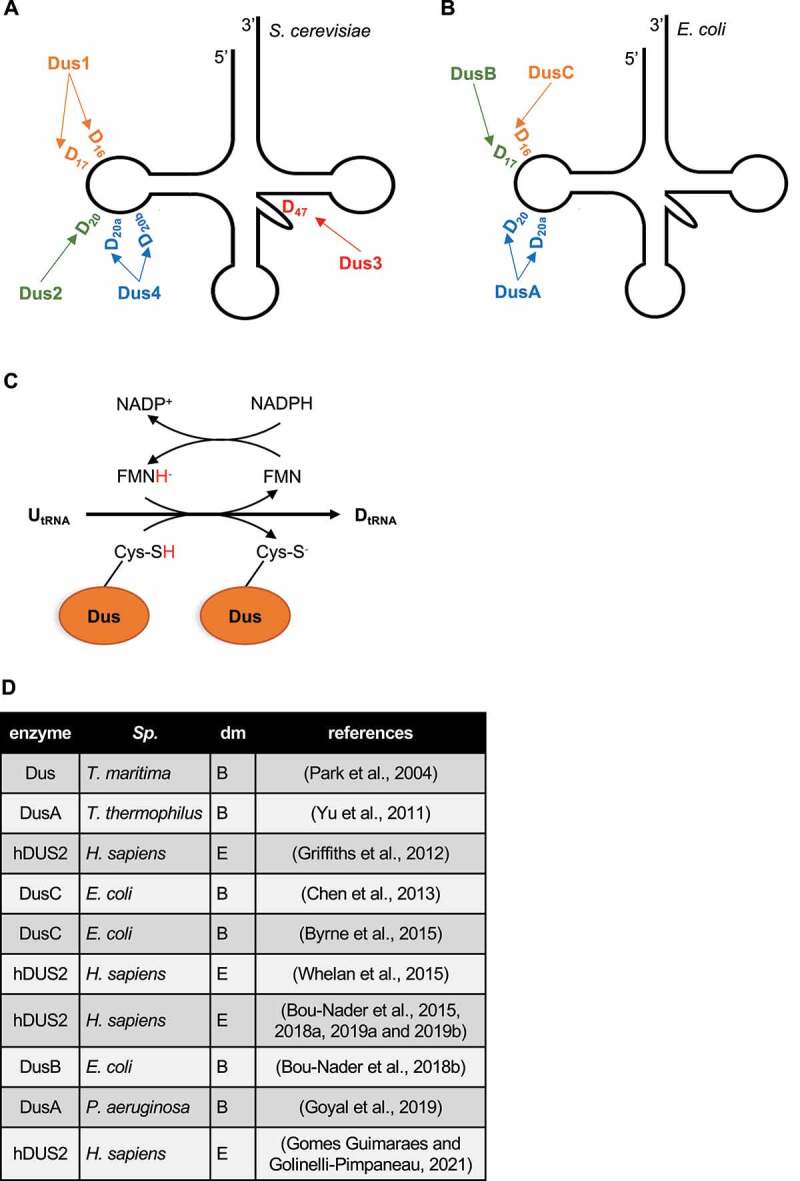
The biology of dihydrouridine.

Based on 602 tRNA sequences from viral, prokaryotic and eukaryotic species, D is the second most prevalent tRNA modification (925 counts) after pseudouridine (1,164 counts) [43]. The dihydrouridylated positions include the canonical D_16_, D_17_, D_20_, D_20a_, D_20b_ and D_47_ (Fig. 4A) and the rare non-canonical D_14_, D_17a_, D_21_ and D_48_. Among the most frequent modified positions are D_20_ and D_16_ both positioned in the eponym *D-loop* that has a pivotal role in the establishment of secondary and tertiary structures of tRNAs [15,44]. The biochemical specificities of D play a role in the cloverleaf-related tRNA secondary structure and in the L-shaped tRNA tertiary structure that is achieved through D- and T-loops interaction (reviewed in [45]). Although the most important residues for the kissing D/T loops are not dihydrouridines, a compilation of crystal structures highlighted a set of base pairing events where D is involved through various types of interactions (*cis* or *trans* interactions between Watson-Crick, Hoogsteen and sugar edges) [46,47].

**A** Specificity of yeast Dus enzymes for cytoplasmic tRNAs.**B** Specificity of *E. coli* Dus enzymes for tRNAs.**C** Putative enzymatic mechanism of Dus enzymes. Details in the text.**D** Currently available structures of Dus enzymes in different species (*Sp*.) from different domains (dm; B for Bacteria and E for Eukarya). The references are indicated in the right column.

### 3.1 Dihydrouridine in eukaryotes

All eukaryotic tRNA species have been described with at least one dihydrouridine, with the exception of tRNA^selenocysteine^. Particularly, the 18 described cytoplasmic tRNA_i_^Met^ only have D_16_ but no D_17_, D_20_, D_20a_, or D_20b_. In mitochondrial tRNAs (mt-tRNAs), all six canonical D are found, except D_20b_ [43]. Importantly, there are dihydrouridylated mt-tRNAs that are encoded by the mitochondrial genome; at least three mammalian mt-tRNAs are known to have a D_20_ and accordingly, the putative cognate enzyme was shown to localize in human and murine mitochondria [48,49]. It has been shown that a set of mammalian mt-tRNAs have a truncated cloverleaf structure by lacking the D-loop [50,51]. Surprisingly, these tRNAs seem to adopt a functional tertiary structure by establishing unique interactions in a Mg^2+^-dependent manner [52,53].

Phizicky and colleagues identified the first dihydrouridine synthase Dus1 in 2002 in *S. cerevisiae* [54] based on a dihydrouridylation assay using a collection of GST-ORF fusion proteins. Homology searches highlighted three related enzymes encoded in the yeast genome. The specific modification of tRNA^Phe^ by Dus1 and tRNA^Leu^ by Dus2 supported that Dus are substrate-specific enzymes, which was confirmed and expanded in an elegant microarray-based experiment relying on the D-dependent alteration of strands interaction. The resulting tRNA D landscape was U_16_ and U_17_ targeted by Dus1, U_20_ by Dus2, U_20a_ and U_20b_ by Dus4 and the extra D-loop U_47_ by Dus3 (Fig. 4A). The budding yeast quadruple *dus* mutant was shown to be viable and to lack any detectable dihydrouridine [33].

Early on, the D landscape was suspected to expand beyond tRNAs as D was found associated with plant and mammalian histone-bound RNAs [55,56] and detected on an enzymatic digest of rat U5 snRNA [57]. However, this was not further analyzed until recently (see below).

### 3.2 Dihydrouridine in prokaryotes

All canonical D residues (D_16_, D_17_, D_20_, D_20a_, D_20b_) are found in Bacteria. D_47_ is a scarce modification with a unique occurence described so far (tRNA^Met^ of *B. subtilis*), although the position 47 in bacterial tRNAs is a U in almost 90% of the 134 known sequences. D_20b_ is also very uncommon and found on a cyanobacterial tRNA^Glu^ [43]. A peculiarity of the bacterial dihydrouridine landscape is the presence of a unique D on the 23S rRNA in Gram-negative (*E. coli*) and -positive (*M. hominis, C. sporogenes*) bacteria [58–60]. In *E. coli*, D is located at position 2449, a residue located in the highly conserved 23S rRNA central loop of domain V [59–61]. Remarkably, this region is part of the peptidyltransferase center that is also one of the sites of interaction with antibiotics targeting ribosomal activity. However, D_2449_ is dispensable in *E. coli* [62]. The Gram-positive *C. acetobutylicum* is the only known organism to have a D on its 16S rRNA. The modification occurs on position 1211 or 1212 but its function is still unknown [63].

Another type of non-coding RNA known to be dihydrouridylated is the Y RNA (a non-coding RNA involved in RNA degradation) from the γ-proteobacterium *S. typhimurium*. It has a DusA-dependent D and folds in a tRNA-like manner [64].

The de Crécy-Lagard laboratory implemented an *in silico* comparative genomic screen in order to find bacterial *dus* genes. Because no D was ever detected in *P. furiosus*, they assumed that the genome of this organism should not contain any *dus* gene, in contrast to other microorganisms such as *E. coli* or *S. cerevisiae*. By doing so, they found ortholog genes absent in the D-free *P. furiosus* species but present in other D-containing species [65]. This approach led to the identification of three *E. coli* Dus enzymes referred to as DusA, DusB and DusC that have non-redundant activities on tRNAs (Fig. 4B). *E. coli* DusB and DusC are mono-specific proteins that target U_17_ and U_16_, respectively while DusA substrates include U_20_ and U_20a_ [66]. Importantly, this conclusion is not valid for the whole bacterial world as the DusB protein from *Mycoplasma capricolum* was shown to modify U17, U20 and U20a, making it the only known Dus enzyme to modify three different tRNA sites [67].

## 4. First insights into the biological relevance of Dihydrouridine

### 4.1 Implications in human health and development

The isolated tRNA^Phe^ from malignant human tissues was reported to contain more dihydrouridines [68]. D is also present in urine samples and more significantly abundant in the urine of lymphoma patients [69,70]. This is in line with the idea that tumour tissues undergo high turnover of tRNAs that can be quantified and used as a noninvasive biomarker for diagnosis and treatment of cancer [38,71,72]. More generally, D can be considered as a metabolic modulator for a large set of pathological conditions; D is upregulated in the serum of patients with the major form of oral cancer in the world (oral squamous cell carcinoma) [73] and downregulated in the serum of mice bearing breast cancer [74]. D is associated with lethal prostate cancer [75].

At the molecular level, the human DUS2 protein (hDUS2) was shown to act as an inhibitory factor of the interferon-induced protein kinase PKR – whose kinase activity is enhanced in melanomas and colorectal cancers [76]. Similarly, the anti-cancer ginsenoside compound was shown to repress the expression of *hDUS2* in human colorectal cancers cells [77].

To date, the most comprehensive study linking D with cancer was provided by Nakamura and colleagues [78]. *hDUS2* showed a 3-fold overexpression in non-small cell lung carcinomas (NSCLCs) compared to healthy samples. The hDUS2 protein followed the same pattern, localized at endoplasmic reticulum and harbored a C-terminal double-stranded RNA binding motif (DSRM or dsRBD). In addition, an interaction between hDUS2 and the glutamyl-prolyl tRNA synthetase EPRS was reported. Phenotypically, the suppression of the tumor cell growth was observed after siRNA-dependent *hDUS2* depletion and the NSCLCs patients with high levels of hDUS2 showed worse prognostics. The subsequent model was that overexpression of hDUS2 led to the hypermodification of tRNAs and consecutive increase of conformational flexibility. Because hDUS2 interacted with EPRS, they hypothesized that the tRNAs were more promptly charged in NSCLCs, which globally resulted in a more efficient translation although this remains to be established. In addition, increased D level was associated with worse outcomes in several cancers [79,80].

hDUS3 was also shown to be an inhibitor of the regenerative ability of the central nervous system [81]. Strikingly, the dihydrouridine was more abundantly detected during neural development in human embryonic stem cells [82].

### 4.2 Implications in prokaryotic growth

The quantitative detection of dihydrouridine led to the conclusion that psychrophilic bacteria have up to 70% more D on tRNAs than their mesophilic counterparts, which is in contradiction with the general observation that tRNAs of psychrophiles tend to be hypomodified [83]. The psychrophilic organisms that grow between 0°C and 20°C – with an optimum at 15°C – have the necessity to cope with low environmental temperatures, unlike the mesophiles bacteria that live above 20°C. It has been established by NMR that low temperatures tend to stabilize the C2’-*endo* conformation of the dihydrouridine ribose moiety [84,85]. The accumulation of D in psychrophilic prokaryotes could therefore constitute an evolutive adaptation to allow these organisms to maintain the conserved L-shaped conformation of tRNA, despite a growth at very low temperatures that otherwise could be detrimental for tRNA structure and function. More generally, the set of modifications present on a tRNA depends on environmental cues, such as the temperature. In agreement with this idea, the *in vitro* synthesis of dihydrouridine in the hyperthermophilic bacterium *T. thermophilus* is possible on an unmodified tRNA^Phe^ at 60°C but not at 80°C where the tRNA substrate needs to carry other modified nucleosides [86]. At the transcriptional level, the gene coding for the mesophilic *Clostridium botulinum* DusB homolog is downregulated during a heat shock stress at 45°C. In line with the above principles, the bacterium would require less D at high temperatures and would therefore decrease the expression of its cognate enzyme [87]. DusC is also differentially regulated in response to the growth temperature in the thermophilic *Bacillus manusensis* bacterium [88].

An interesting case is found in the Archaea *Methanococcoides burtonii* that has a thermal niche around 2°C and an *in vitro* optimal growth at 23°C. It was noticed that despite its low percentage of modified tRNA nucleotides – only 2%, which is one of the lowest in the living world – this psychrotolerant archaeon has, on average, more than one D residue per tRNA [89]. The fact that an archaeon living at low temperatures and having a limited tRNA epitranscriptome possesses D residues is another clue that D impacts the flexibility of tRNAs.

Finally, D was also shown to be dramatically affected when the *Lactobacillus agilis* gastrointestinal bacterium is grown on an alternative energy source, underlying again the dynamic regulation of this modification [90].

## 5. Structure-function analyses of the Dus enzymes

Dus are flavin-dependent enzymes that function similarly to the dihydroorotate and dihydropyrimidine dehydrogenases. Based on kinetics and structural data, an FMN- and NADP-dependent enzymatic mechanism has been proposed [91–93]. The reductive half-reaction is initiated with NADPH binding to the Dus enzyme. The NADPH transfers a hydride to the Dus-bound flavin prosthetic group (FMN to FMNH^−^), which reduces the enzyme. The dissociation of NADP results in a free reduced enzyme that binds the tRNA. In the oxidative half reaction, the nucleophilic C6 of uridine is reduced. The second hydride transfer to C5 likely occurs through the oxidation of a highly conserved Dus cysteine residue (Cys) that argues in favour of an evolutionary conserved mechanism (Fig. 4C) [94,95]. Interestingly, the reduced Dus enzyme has a very slow oxidative half-reaction when an *in vitro* transcribed tRNA is used as a substrate, contrary to a purified tRNA (from a *dus*- mutant). This strongly suggests that a tRNA already bearing modifications is the genuine substrate and that an ordering of modifications may exist *in vivo* [92].

**Figure 5.**
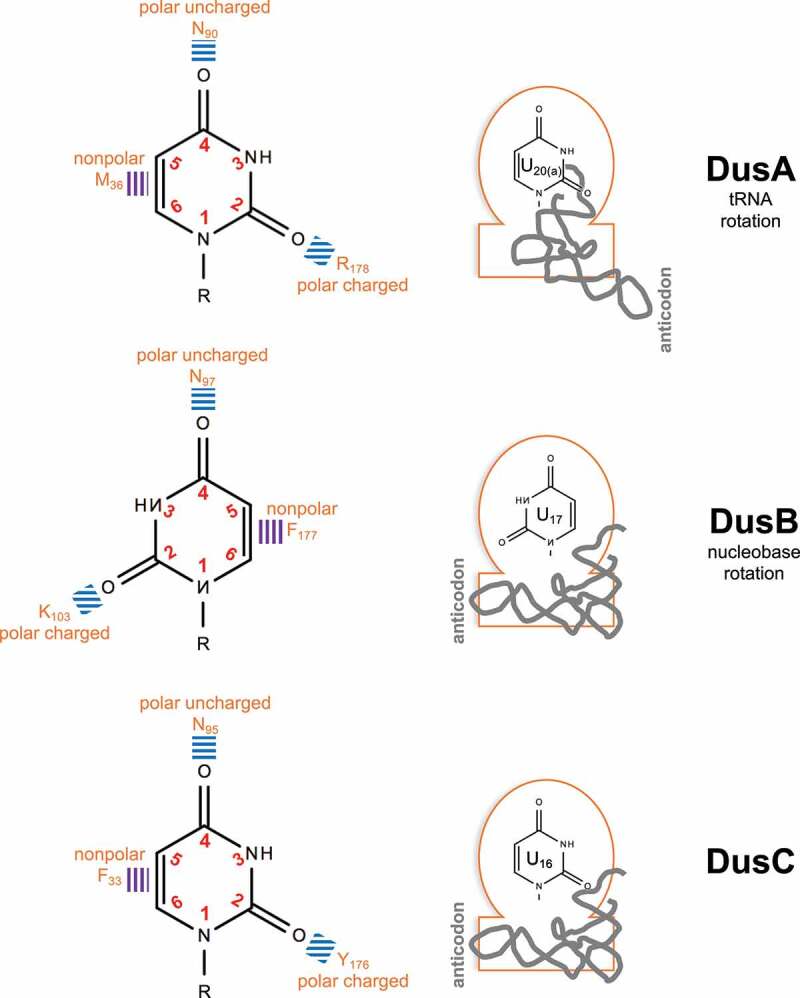
Molecular strategies of bacterial Dus enzymes for substrate specificities.

To date, six Dus enzymes structures have been published (Fig. 4D). The seminal crystallographic structure of an unknown FMN-binding protein in *T. maritima* revealed an oxidoreductase enzyme with two domains; an N-terminal TIM barrel and a C-terminal helical domain [96]. Later, this enzyme was referred as a dihydrouridine synthase. The *T. thermophilus* DusA crystal highlighted the same general structure [93]. Moreover, the FMN cofactor (flavin mononucleotide) was captured in a positively charged groove at the center of the N-terminal domain corresponding to the catalytic site. DusA-tRNA^Phe^ complex revealed that DusA interacts with the D-stem loop, the anticodon stem loop and the T-stem loop of tRNA and that the D-loop but not the D-stem is strongly distorted when DusA is bound. The third published bacterial Dus structure was DusC from *E. coli* that also displays a two-domain conformation with an N-terminal catalytic domain and a C-terminal RNA binding domain [97,98]. The structural similarities between *T. thermophilus* DusA and *E. coli* DusC led to the hypothesis that they share the same catalytic mechanism. Remarkably, notable structural dissimilarities were discovered by comparing the bacterial DusA (targeting tRNA-U_20_ and _20a_) and DusC (targeting tRNA-U_16_). Both enzymes adopted the same general fold – while having different substrate specificities – but bound and recognized the tRNA in different orientations. The tRNA binding differed by a 160° rotation that resulted in the proper integration of the targeted uridine in the catalytic pocket. This trademark way of catalyzing a reaction is unique in RNA enzymology and is achieved through specific *binding signatures*. According to its target, each Dus enzyme has a cluster of amino acids – that is phylogenetically conserved in Bacteria – that defines the docking of tRNA to allow the reduction of a specific uridine [97]. The missing DusB structure was provided by Hamdane and colleagues [66]. Even though the crystal was incomplete, it was concluded that *E. coli* DusB adopted the same overall structure with an N-terminal TIM barrel fold carrying the catalytic function and a C-terminal helical domain. The tRNA docking in DusB was similar to the one of DusC, which makes sense since DusB and C modify neighboring nucleotides (17 and 16). However, a major difference between DusB and C relied on the positioning of the nucleobase into the catalytic center. Reversed polar and nonpolar amino acids in the catalytic pocket of DusB led to the 180 degrees rotation of the nucleobase that is targeted for reduction. In conclusion, diversification of bacterial Dus specificities was made possible through two astonishing strategies; nucleobase rotation or tRNA docking rotation (Fig. 5). More recently, the DusA structure from *P. aeruginosa* was resolved by combining *in vitro* and *in silico* methods [99].

**Left panel**, interaction of the U nucleobase (in the context of the RNA chain (R)) with Dus amino acids (orange: F for Phe, N for Asn, K for Lys and Y for Tyr) through ionic (blue lines) or hydrophobic (purple lines) interactions. In the DusB catalytic pocket, the rotation of the U nucleobase (180°) is observed in comparison to the DusA and C counterparts. **Right panel**, schematic representations of the Dus enzymes (orange line) with the N-terminal nucleobase-containing catalytic domain (round) and the C-terminal RNA binding domain (rectangle). The tRNA (gray line) docking is similar in DusB and C but differs by a rotation (160°) in DusA. These strategies (tRNA or nucleobase rotations) allow the unvariable targeting of a specific uridine.

The only available eukaryotic Dus structure is the human DUS2. hDUS2 is particularly important because it is associated with pulmonary carcinogenesis and, unlike other Dus enzymes, harbors a dsRBD. This domain turns out to be conserved in animals (mammals, amphibia, flatworms, nematodes, insects) [94]. The sequence similarity is quite low between hDUS2 and bacterial Dus or even yeast Dus2, suggesting a potential novel strategy for the substrate recognition [100]. The analogy between bacterial Dus and hDUS2 includes an N-terminal catalytic domain folded in a TIM barrel, an interaction of the catalytic domain with FMN, a high sequence conservation of the residues required in the active site – including the cysteine as an H^−^ donor and the presence of a central helical domain. The C-terminal dsRBD domain was shown to be necessary but not sufficient for D_20_ synthesis on yeast tRNA extracted from a *∆dus2* strain. Furthermore, this domain was suggested to serve as primary tRNA binding site, before the canonical helical domain [101]. Using a dsRBD for tRNA recognition was never reported before, making hDUS2 the only known tRNA modifying enzyme to adopt this strategy. This unexpected feature was recently clarified by showing that the helical domain of hDus2 was less electropositive than its yeast orthologue partly because of the absence of two lysine residues, which played a role in the emergence of a new tRNA binding mode [102,103].

## 6. New high-resolution methods to decipher the transcriptome-wide distribution of Dihydrouridine

As indicated above, NaBH_4_ or OH^−^ treatments result in an abasic site (nucleobase-free ribose) leading to the cleavage of the RNA chain with aniline treatment [34,35]. This led to the development of AlkAniline-Seq, a method relying on the chemistry-based enrichment of sequencing libraries with fragments containing certain modifications including D [104,105]. While AlkAniline-Seq responded to the presence of D, the signal strength was considerably lower than for other marks including m^7^G. This is likely due to the incomplete formation of abasic sites at D position following limited alkaline hydrolysis.

On a completely different basis, RNA-mediated activity-based protein profiling (RNABPP) relies on metabolic RNA labelling, mRNA interactome capture and quantitative proteomics. In this approach, a 5-halopyrimidine (typically 5-FUrd, 5-fluorouridine for DUS3L) is incorporated in RNA and form stable RNA-protein adducts with the modifying enzyme, allowing RNA interactome capture [106]. These experiments confirmed that U46-48 in the tRNA variable loop is the major substrate of DUS3L. Notably, the approach also revealed DUS3L crosslinking peaks on non-tRNA substrates including mature mRNAs [106]. These data are reminiscent of a previous work reporting that in cardiomyocytes, the human DUS3 homolog was shown to interact with polyA^+^ RNAs, raising the possibility of mRNAs hDUS3-specific modification in this specialized cell type [107].

We recently introduced Rho-seq to globally detect the presence of D residues on RNA [108]. In a two-step protocol, total RNA is first incubated with NaBH_4_ and then covalently bound by the rhodamine fluorophore (Rho). From there, the Rho-seq protocol unfolds as follows; (I) RNA extraction from WT and *∆4* strains (lacking the four *dus* genes), (II) Rho+ and mock-treatments, (III) ribodepletion, (IV) cDNA synthesis and library preparation, (V) strand-specific deep-sequencing, (VI) data analysis by implementation of a multifactorial analysis. The detailed protocol is available in a separate publication [109].

Rho-seq provided the first transcriptome-wide Dus1 to 4-specific repertoire of the distribution of D and highlighted that D is an integral component of yeast and human mRNAs, though at low level. These data also provided a framework for the previous findings that DUS1 and DUS3 cross-link to mRNAs in both yeast and human [110,111].

The absence of detected D-sites on the bacterial coding transcriptome suggests that mRNA dihydrouridylation may be a eukaryotic-specific mechanism although this requires additional support [108].

A set of *in silico* tools were developed to predict the presence of D on various RNA types. These computational predictions need however to consider a larger set of proven D-containing sequences to build more objective tools [112–115].

Finally, the detection of modified nucleotides with the Oxford Nanopore Technologies is still in its infancy but further developments will likely confirm the seminal detection of D on *E. coli* tRNAs through the nanoscale pore [116].

## 7. Is dihydrouridine an adaptative mark regulating translation?

Similarly to other mRNA modifications, the detected D-sites could be relevant in a specific physiological context. Two previously reported cases are illustrated in the literature: induced pseudouridylation upon heat shock and widespread methylation during the meiotic programme [117,118]. We found that the deletion of *dus3* in fission yeast specifically affects meiotic chromosome segregation and the translation of a set of proteins including tubulins while the mitotic cell cycle is barely affected [108].

Ribosome profiling and proteomics revealed that a dihydrouridylated mRNA can be translated but that the modification affects this process, which may result in modulation of the translational speed and ribosome stalling [108]. In the late sixties, several teams investigated the *in vitro* coding properties of D-containing oligoribonucleotides. Despite the technical boundaries encountered at that time, they concluded that the presence of D resulted in the loss of coding ability. Rottman and Cerutti showed that a ribopolymer carrying 4.2% of D (and 95.8% of U) lost up to 60% of Phe(UUU) residues incorporation into the protein [119]. Another study highlighted the complete loss of ability for GUD, GDU and GDD trinucleotides to code for Val (GUU) [120]. The conclusion was the same for the dihydrouridylation of the AUG (Met) codon [121,122]. These were the first data supporting that D-containing mRNAs could be translationally repressed.

The mRNAs encoding several subunits of the CCT (chaperonin-containing tailless complex polypeptide 1) complex, which is required for the folding of newly synthesized tubulin and actin proteins [123], contain D. A possibility to investigate is that the D modification modulates the speed of translation to allow co-translational folding. Interestingly, Dus2 was shown to help in the detoxification of the amyloid-β peptides which form aggregates in the Alzheimer’s disease [124].

As stated above, the alterations of the expression level of some Dus enzymes and the D landscape in cancers will be the topic of additional investigations to understand if translation is affected and to test if this is mediated by modified mRNAs and/or tRNAs.

## Perspectives

It took almost 40 years from the first detection of dihydrouridine within tRNAs to the discovery of Dus enzymes, and 20 more years to expand the D landscape to the coding RNA world. Recent advances open up several interesting research directions.

A peculiarity of *E. coli* is the 23S-D_2449_ deposition is independent of the *dusA-B-C* genes [108]. To our knowledge, this is the first experimental evidence suggesting that D could be deposited by a *non-canonical* dihydrouridine synthase. In Bacteria, uracil can be reduced to dihydrouracil by the dihydropyrimidine dehydrogenase complex formed by PreA and PreT [125]. Noticeably, the dihydropyrimidine dehydrogenase is of clinical importance in human because deficiency in this enzyme (DPYD in *H. sapiens*) leads to a severe sensitivity to the administration of 5-fluorouracil, an agent used widely to treat cancer [126].

The catalytic activity of the DPYD enzyme is reversible, suggesting the immediate possibility that Dus enzymes may also function backward as D eraser to restore uracil from D in some circumstances. A proof of concept supporting this possibility was already reported [92].

It will also be of interest to explore the D landscape in plants where it is predicted to be present on mRNAs as well [127]. Interestingly, D could be a developmentally regulated modification in *A. thaliana* where the *DUS* orthologs are expressed at low level in rosette leaves and apex tissues, unlike other RNA modifying enzyme genes [128].

Deciphering the synthesis of a carboxypropylated dihydrouridine (acp^3^D) from D in *T. brucei* is another important perspective to unravel the biology of this complex modification [129].

Another interesting possibility discussed above is the capacity for D to alter the recognition of RNA by interacting proteins. While the possibility of D readers will be investigated by classical unbiased approaches, a pilot study predicted 20 RNA binding-proteins (RBPs) to interact with D [114]. Interestingly, these RBPs are enriched for the alternative mRNA splicing process, including the SRSF9, SFPQ and ESRP2 splicing factors.

The abundant, simple yet fascinating D modification is only about to reveal its importance.

## Supplementary Material

Supplemental Material

## Additional references from Figures:

Bou-Nader et al. [130], Guimaraes and Golinelli-Pimpaneau [131], Molinaro et al. [132], Whelan et al. [133].

Dalluge et al. [134], Davis et al. [135], Deb et al. [136], Deslauriers et al. [85], Formoso and Tinoco, Jack et al. [137], Quigley and Rich [138], Rohrer and Sundaralingam [139], Suck et al. [140], Suck et al. [141], Sundaralingam et al. [142], Westhof et al. [143], Westhof and Sundaralingam [144], Woo et al. [145], [146].
